# Multi-Generational Drinking of Bottled Low Mineral Water Impairs Bone Quality in Female Rats

**DOI:** 10.1371/journal.pone.0121995

**Published:** 2015-03-24

**Authors:** Zhiqun Qiu, Yao Tan, Hui Zeng, Lingqiao Wang, Dahua Wang, Jiaohua Luo, Liang Zhang, Yujing Huang, Ji-an Chen, Weiqun Shu

**Affiliations:** 1 Department of Environmental Hygiene, College of Preventive Medicine, Third Military Medical University, Chongqing, China; 2 Department of Health Education, College of Preventive Medicine, Third Military Medical University, Chongqing, China; University of Milan, ITALY

## Abstract

**Background:**

Because of reproductions and hormone changes, females are more sensitive to bone mineral loss during their lifetime. Bottled water has become more popular in recent years, and a large number of products are low mineral water. However, research on the effects of drinking bottled low mineral water on bone health is sparse.

**Objective:**

To elucidate the skeletal effects of multi-generational bottled water drinking in female rats.

**Methods:**

Rats continuously drank tap water (TW), bottled natural water (bNW), bottled mineralized water (bMW), or bottled purified water (bPW) for three generations.

**Results:**

The maximum deflection, elastic deflection, and ultimate strain of the femoral diaphysis in the bNW, bMW, and bPW groups and the fracture strain in the bNW and bMW groups were significantly decreased. The tibiae calcium levels in both the bNW and bPW groups were significantly lower than that in the TW group. The tibiae and teeth magnesium levels in both the bNW and bPW groups were significantly lower than those in the TW group. The collagen turnover markers PICP (in both bNW and bPW groups) were significantly lower than that in the TW group. In all three low mineral water groups, the 1,25-dihydroxy-vitamin D levels were significantly lower than those in the TW group.

**Conclusion:**

Long-term drinking of low mineral water may disturb bone metabolism and biochemical properties and therefore weaken biomechanical bone properties in females. Drinking tap water, which contains adequate minerals, was found to be better for bone health. To our knowledge, this is the first report on drinking bottled low mineral water and female bone quality on three generation model.

## Introduction

Over the past decade, the consumption of bottled water has increased consistently because of the perceived risks of tap water, the perceived safety of bottled water, the preferred taste and the convenience of drinking bottled water [1]. There are different types of bottled water: purified water, artificial mineralized water, spring water and sparkling water [2]. Purified water has very low total hardness (TH) and total dissolved solids (TDS) because it is produced by distillation, deionization, reverse osmosis, or other suitable processes (IBWA). To increase the total hardness and improve the taste of bottled purified water, producers add some minerals to bottled purified water, which is sold in markets as bottled artificial mineralized water. Another highly consumed bottled water is natural water, which is minimally processed water that usually maintains the characteristics of the water source (IBWA). Because bottled water has become a main type of drinking water, second only to tap water, bottled water’s health impacts need to be assessed [3]. However, there are few reports about the multi-generational long-term health effects of these types of water in human and animal.

The effect of water total hardness on cardiovascular disease [4, 5], alcoholic liver disease [6] and the bone [7–9] were reported. Total hardness primarily includes Ca^+2^ and Mg^+2^, while Sr^+2^, Fe^+2^ and Mn^+2^ also usually contribute to an insignificant degree [10]. Bone is the main reservoir for minerals and plays an important role in regulating mineral homeostasis [11]. Bicarbonate and magnesium from drinking water could affect bone health by lowering bone resorption [9, 12]. The lack of calcium induces a deficiency in the bone reservoirs resulting in bone brittleness and an increased risk of fractures (osteoporosis) [13]. Increased bone mineral density and reduced urinary calcium excretion were observed following the administration of potassium citrate [14]. High sodium intake is associated with high calcium excretion, and causes reduction in the activity of biomarkers of bone formation and resorption, and higher rate of bone mineral loss in postmenopausal women [15]. The acid-base conditions in the body can also influence mineral homeostasis [16]. And one study showed that drinking lower pH water increased the risk of forearm fractures [12]. Two other studies revealed that natural mineral water represents a substantial alkaline load and may influence calcium homeostasis and bone remodeling [17, 18].

Although the influence of water hardness, TDS, mineral elements and pH on bone health have been reported individually, no experimental studies have assessed the effects of drinking bottled low mineral water, for which all these parameters are low, on bone health. And in term of the effects of drinking water on human health mostly need long term observation, the aim of the current study was to observe the effect of multi-generational drinking bottled low mineral water on bone health of female which being charged by pregnancies, lactations and menopause during their lifetime, were susceptible on bone loss [19], in order to understand and prevent the potential hazard effects of low mineral water on sensitive populations.

## Methods

### Ethics Statement

The animal work was approved by the Third Military Medical University Animal Ethics Committee. Animals were sacrificed using intraperitoneal injections of 10% chloral hydrate.

### Drinking Water samples

Tap water (TW), bottled natural water (bNW), bottled mineralized water (bMW) and bottled purified water (bPW) were used in this study. TW was collected from the municipal water supply in Chongqing city in Southwest China. The bNW, bMW and bPW were bought from the market. Each bottled water type had the same manufacture date. The qualities of 4 kind of water were measured according to standard drinking water examination methods (GB/T5750-2006 and GB19298-2003, China). The sodium (Na), potassium (K), calcium (Ca) and magnesium (Mg) content of these water samples was determined using inductively coupled plasma atomic emission spectrometry (ICP-AES, Spectro Ciros CCD, Germany).

### Animal study

This experiment was carried out based modified on an OECD Two-generation study (OECD416) and China Reproduction Toxicity Study GB15193.15–2003. Four-week-old Sprague-Dawley rats were randomly divided into four groups to form the F0 generation of rats drinking four water types: tap water (TW group), bottled natural water (bNW group), bottled mineralized water (bMW group) and bottled purified water (bPW group), which were administered freely. There were 30 female rats and 15 male rats in each group. During the premating periods, the F0, F1and F2 generation animals were weighed individually once a week. At 17 weeks of the age, two females were paired with one male (2:1) from the same treatment group; vaginal smears were examined for the presence of sperm. The presence of spermatozoa in the vaginal smear was considered day zero of pregnancy. Each pregnant female rat was caged separately. The day of birth was identified as postnatal day (PND) 0 or lactation day (LD) 0. The body weight and food consumption of males and females were measured weekly.

Thirty female and fifteen male F1-generation rats in each group were chosen to drink the same water as their parents after weaning and were chosen to mate to generate F2-generation offspring at 17 weeks of age using the method used in the F0 generation. Thirty female F2-generation rats in each group were chosen to continue drinking the same water as their parents until 10 months of age.

The rats were maintained under standard laboratory conditions in an air-conditioned room. Lighting was maintained in a reverse 12-hour light/dark cycle, temperature was kept at 22±2°C, and humidity was constant at 50±10% (relative humidity).

The F2-generation female rats were euthanized at 10 months of age using intraperitoneal injections of 10% chloral hydrate (3 ml/kg body weight). Blood was immediately collected via cardiac puncture for an acid-base status test and centrifuged to obtain serum, which was stored at −80°C until analysis. The right tibiae, both femora and incisor teeth were dissected and soft tissue was removed. The isolated bone and teeth were wrapped in saline-soaked gauze and stored at −20°C until analysis.

### Biomechanical measurements

A three-point bending test was performed to estimate the biomechanical properties of the femoral diaphysis. All right femora, which were fully thawed, were placed horizontally on two supporting bars; the femora were then loaded at the mid-diaphysis in the A–P plane until fracture, as described in detail elsewhere [20, 21]. Bone structure property parameters, including ultimate load (N), elastic load (N), maximum deflection (mm), and elastic deflection (mm), and material property parameters, including Young modulus (MPa), ultimate stress (MPa), ultimate strain (mm/mm), and fracture strain (mm/mm), were detected by an Instron 1011 universal material testing machine (Instron, MA, USA). The tensile loading speed for all tests was 2 mm/s.

### Bone Density Measurements

Bone mineral density (BMD) was determined using dual-energy x-ray absorptiometry (DXA, Lunar PIXImusII mouse densitometer; GE LUNAR Corp.). All right femora were fully thawed before scanning. To mimic the soft tissue present in vivo, the bones were placed in a physiological saline solution during the exam. This apparatus is commonly used in small animals [22].

### Bone mineral content measurements

The bone mineral content was determined in the tibiae and teeth. All right tibiae and teeth (10 in each group, respectively) were dried to a constant weight at 105±5°C (teeth, 2 h; tibiae, 3 h) and then ground in a mortar. Next, 0.1-g samples were put directly into Teflon vessels and digested by applying wet microwave digestion. The samples were digested with 8 ml of HNO_3_- 30% H_2_O_2_ (3:1)in a microwave dissolver (Multiwave 3000, Anton Paar) at a 0.5 bar/s heating rate. The samples were then transferred into 50-mL measuring flasks and filled to volume [23]. Afterwards, calcium and magnesium content in the specimens were measured by atomic absorption spectrophotometry (TAS-983 atomic absorption spectrophotometers, Purkinje General, China). Standard reference material calcium (GSB04-1720-2004) and magnesium (GSB04-1735-2004) were used throughout the study to validate the analytical technique.

### Serum biomarker analyses

Levels of serum bone specific alkaline phosphates (BALP), carboxy-terminal propeptide of type I procollagen (PICP) and cross-linked telopeptide of type I collagen (ICTP) were quantified for 10 female rats in each group using commercial enzyme-linked immunosorbent assay (ELISA) kits (Pierce Chemical, Rockford, IL, USA) according to the manufacturer’s instructions. 1, 25-Dihydroxyvitamin D levels in serum were measured using commercial enzyme-linked immunosorbent assay (ELISA) kits (Shanghai Bogoo Biotechnology Co., Ltd, Shanghai, China) according to the manufacturer’s instructions.

### Statistical analysis

SPSS 18.00 software for Windows (SPSS Inc. Chicago, IL, USA) was used for all statistical analysis. Parameters are expressed as Mean±SEM. Statistical comparisons were performed using one-way ANOVA followed by Bonferroni's correction for multiple comparisons. P values of 0.05 or smaller were considered statistically significant.

## Results

### Characters of water

All 4 types water was met the water quality standard according to 36 organoleptic, physical and chemicals, toxicological parameters (data not show). And the main differences were the level of total hardness (as CaCO_3_), total dissolved solids and mineral elements. The calcium, magnesium and sodium content in tap water were obviously higher than that of the other three bottled water types (Table 1). More characteristics of the 4 types water were shown at S1 Table.

**Table 1:** Main mineral element contents and pH values of the four types water.

	TW	bNW	bMW	bPW
pH value	7.57	7.55	6.80	6.80
Total dissolved solids (mg/L)	229	87.2	10.9	1.2
Total hardness(CaCO_3,_ mg/L)	200.3	69.6	2.3	0.8
Calcium (mg/L)	52.9	10.6	0.02	0.04
Magnesium (mg/L)	12.7	9.4	0.4	0.02
Sodium (mg/L)	12.4	9.0	0.1	0.1
Potassium (mg/L)	2.5	3.8	3.4	≤0.5

### Femur biomechanical properties

**Fig 1:**
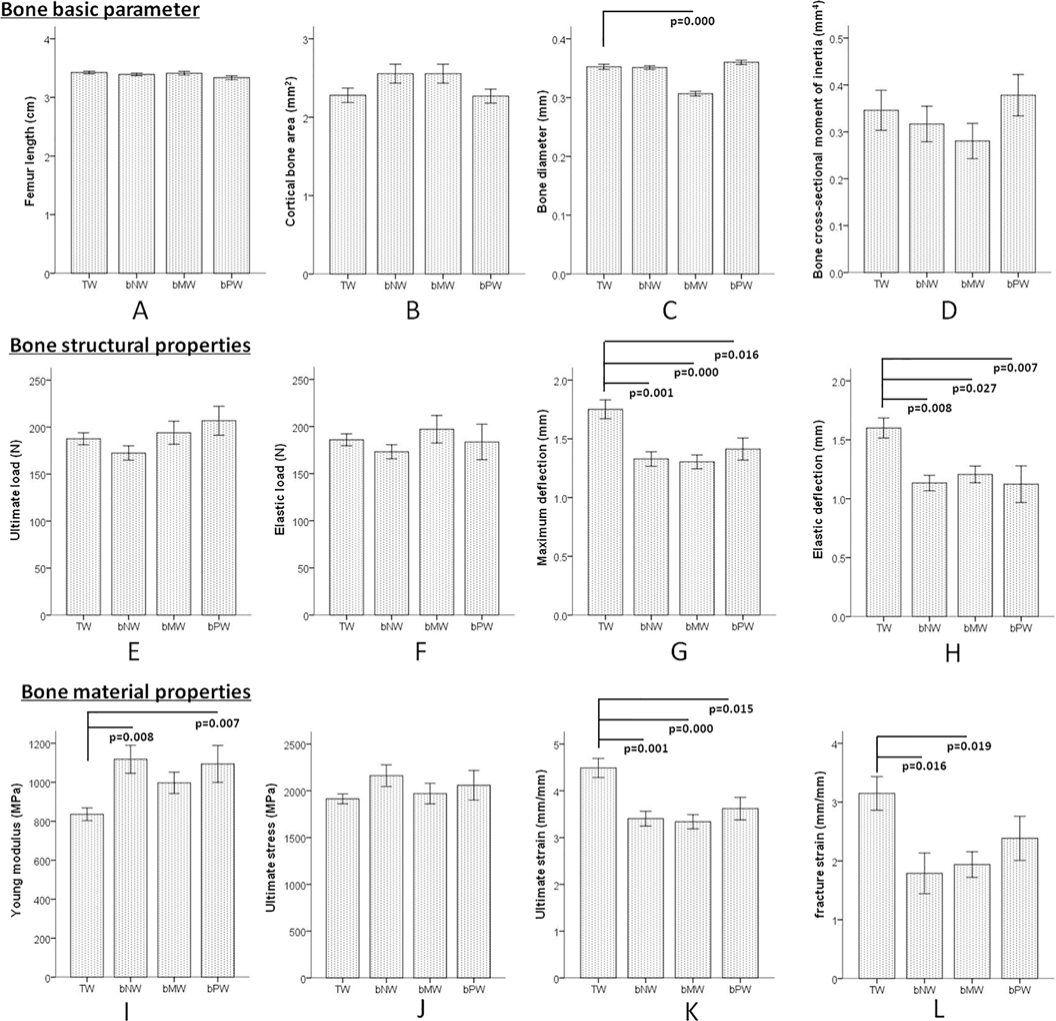
Femur biomechanical properties, according to the three-point bending test, of F2 female rats after three generations of continuous drinking of four water types. The basic bone parameters, including length, cortical bone area, bone diameter and bone cross-sectional moment of inertia, are shown in A, B, C, and D, respectively; the bone structural properties, including ultimate load, elastic load, maximum deflection of femoral diaphysis and elastic deflection, are shown in E, F, G, H, respectively; the bone material properties, including Young modulus, ultimate stress, ultimate strain and fracture strain, are shown in I, J, K, L, respectively. TW: Tap water, bNW: bottled natural water, bMW: bottled mineralized water, bPW: bottled purified water.

The three-point bending test is the classic method used to evaluate the biomechanical properties of bone. Fig 1 shows the results for the bones’ biomechanical properties. Length, cortical bone area and bone cross-sectional moment of inertia were not significantly different between the four groups (Fig 1A, 1B and 1D), while the bone diameter of the femur in the bMW group was significantly lower than that in the TW group (Fig 1C). Regarding the structural bone properties, compared to the TW group, both the maximum deflection of the femoral diaphysis and the elastic deflection in the bNW, bMW and bPW groups were evidently decreased (Fig 1G and 1H), while the ultimate load and elastic load among the 4 groups were not significantly different (Fig 1E and 1F). For bone material properties, the Young modulus in both the bNW and bPW groups was significantly higher than that in the TW group (Fig 1I). The ultimate strain in the bNW, bMW and bPW groups was significantly lower than that in the TW group (Fig 1K). The fracture strain in both the bMW and bNW groups was significantly decreased compared to that in the TW group (Fig 1L). There was no significant difference in ultimate stress among the four groups (Fig 1J).

### Bone mineral contents and bone mineral densities

**Fig 2:**
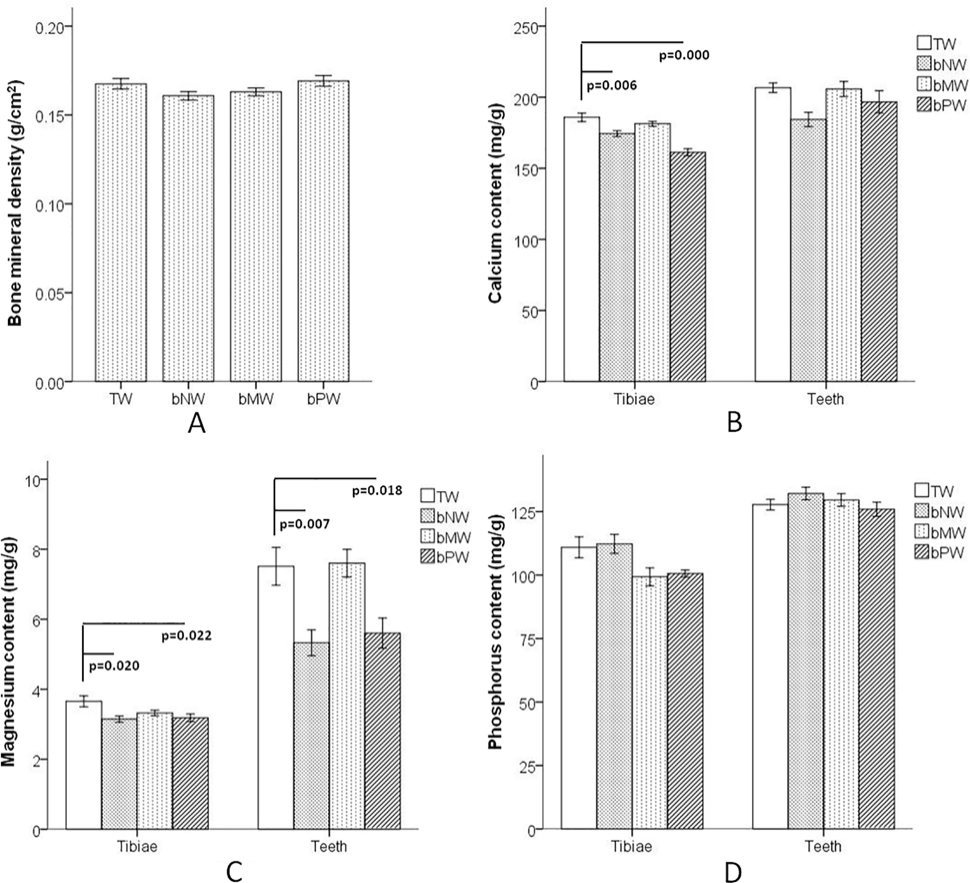
Bone mineral density of femora and bone mineral content of tibiae and teeth of F2 generation female rats after three generations of continuously drinking the four water types. A shows the bone mineral density of femora, B shows the calcium content of both the tibiae and teeth, and C shows the magnesium content of both the tibiae and teeth. TW: Tap water, bNW: bottled natural water, bMW: bottled mineralized water, bPW: bottled purified water.

Although there was no significant difference in the bone mineral density of the femora tested by DXA among the 4 groups (Fig 2A), the main mineral contents (calcium and magnesium) in bone and teeth were altered after drinking different water types (Fig 2B and 2C). Calcium in the tibiae of both the bNW and bPW groups was significantly lower than that in the TW group (Fig 2B). Magnesium in both the tibiae and teeth of both the bNW and bPW groups was significantly lower than that in the TW group (Fig 2C). There were no differences in the phosphorus content of the tibiae or teeth among the 4 groups (Fig 2D).

### Biochemical indicators of bone metabolism

**Fig 3:**
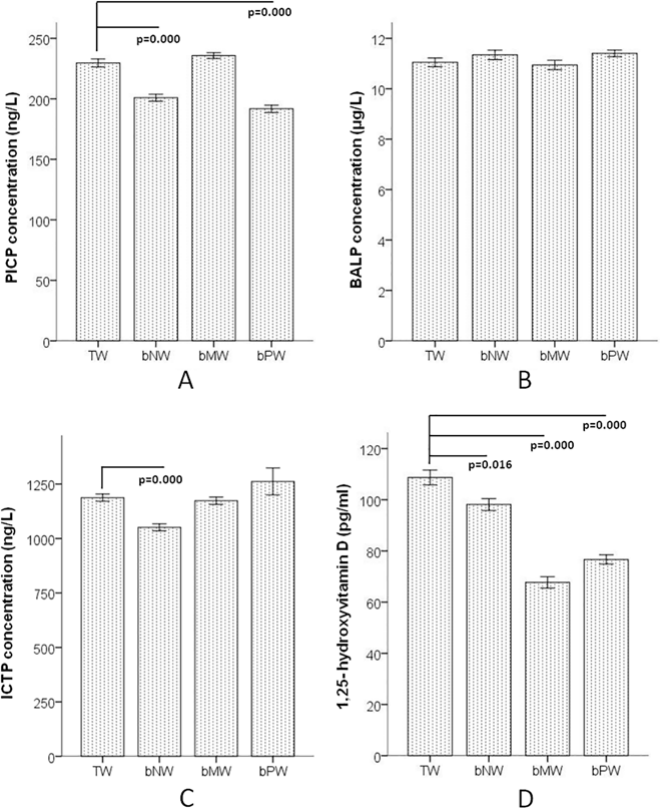
Biochemical indicators of bone metabolism in F2 generation female rats after 3 generations of continuously drinking the 4 water types. A shows the level of PICP, B shows the level of BALP, C shows the level of ICTP and D shows the level of 1,25-dihydroxyvitamin D in serum. TW: Tap water, bNW: bottled natural water, bMW: bottled mineralized water, bPW: bottled purified water.

The levels of collagen turnover markers PICP and ICTP and bone formation marker BALP in the serum were selected to indicate bone metabolism. PICP in both the bNW and bPW groups were significantly lower than that in the TW group (Fig 3A) and ICTP also has a decreasing trend in the bPW group compared to the TW group (Fig 3C), while the serum BALP concentrations among the 4 groups were not significantly different (Fig 3B). The level of 1,25-dihydroxyvitamin D in all three low mineral water groups (bNW, bMW and bPW) was significantly lower than that in the TW group (Fig 3D).

## Discussion

Drinking water is an important supply of mineral elements for human skeletal health except food [24–26], and mineral elements in drinking water can influence bone metabolism and turnover [27]. All four types water meet the drinking water standards in China (GB5749-2006 and GB 19298–2003). The total hardness and total dissolved solids of tap water were in the medium level of that in China. Our results indicated that product process for three bottled water removed a large portion of mineral elements from source water.

The detection of biomechanical properties is indispensable for evaluating bone quality. Our results showed that elastic deflection and maximum deflection in the bNW, bMW and bPW groups were lower than the same parameters for the TW group; therefore, the ability of bone to resist external forces and deformation was weaker in the bNW, bMW and bPW groups. These results reveal that the matrix collagen content in bone might be lower and that the brittleness of bone might be higher after drinking bottled low mineralized water. The Young's modulus, stress, and strain reflect intrinsic bone properties; these parameters indicate the mechanical properties of bone components and their spatial structure, which are mainly effected by bone mineral density and trabecular orientation, regardless of the size of the bone [28]. Ultimate strain and fracture strain were both significantly decreased after drinking bottled mineralized water and natural water, and only ultimate strain decreased after drinking bottled purified water. These results suggest that the rats drinking bottled low mineralized water had weaker bone tenacity and could not resist as strongly against external forces as the rats drinking tap water. This result was in accordance with the structural mechanics results. The Young's modulus was obviously increased only after drinking bottled natural water compared to tap water. This result hinted that this difference may occur because of the complicated changes in bone turnover parameters. A previous study demonstrated that the Young's modulus increased after rats consumed cadmium from drinking water [29].

The skeleton stores calcium and regulates blood calcium levels. Bone phosphorus can adjust the acid-base balance of body fluids and coeffect with calcium to promote bone matrix synthesis and bone mineral deposition [30]. Magnesium maintains and promotes bone growth [31]. Although there were no differences in BMD among the four groups, the calcium and magnesium contents in the tibiae and teeth showed disparities. Some animal experiments and clinical research have demonstrated that BMD is not always fully consistent with bone mineral content [32, 33]. The calcium levels in the tibiae and teeth of the TW group were the highest, while the calcium levels in the tibiae of the bPW group were the lowest among the 4 groups. This result was consistent with the calcium content of the drinking water types. For the content of calcium of tibiae in bNW and bPW group were lower than other two groups but that of teeth were not, the reason might be that inadequate intake of calcium aroused bone calcium into blood to keep the balance of blood calcium level, but the calcium in teeth would not take part in this mechanism. And bone calcium is more sensitive with inadequate intake than teeth calcium.

The circulating collagen fragment PICP and ICTP concentrations were reliable biochemical markers of bone remodeling pattern. Serum PICP in both the bNW and bPW group and ICTP in bNW were significantly lower than that in the TW group. This result revealed that long-term consumption of bottled low mineral water inhibited bone turnover. Although there was no significant difference in the bone mineral density (BMD) among the 4 groups, the calcium level in the tibiae of both the bNW and bPW groups was obviously lower than that of the other groups. According to previous studies, biochemical indicators, such as PICP, are more sensitive than BMD for detecting bone metabolism [34, 35].

1,25-Dihydroxyvitamin D is the active form of vitamin D and is primarily generated in the kidney from 25-hydroxyvitamin D. 1,25-Dihydroxyvitamin D circulates in lower concentrations than 25-hydroxyvitamin D but has much greater affinity for the vitamin D receptor and is more biologically potent [36]. 1,25-Dihydroxyvitamin D enhances intestinal calcium absorption in the small intestine and induces preosteoclasts to become mature osteoclasts. Osteoclasts remove calcium and phosphorus from the bone and maintain calcium and phosphorus levels in the blood. Adequate calcium and phosphorus levels promote the mineralization of the skeleton [37]. The 1,25-dihydroxyvitamin D levels in all three low mineral water groups were significantly lower than that in the TW group. This result revealed that the long-term consumption of bottled low mineral water might disturbed the mineralization of the skeleton according to inhibit the vitamin D activation. One previous study showed a doubling of the risk of hip fracture in postmenopausal women with low serum 1,25-dihydroxyvitamin D[38]. The serum 1,25-dihydroxyvitamin D concentration compensatory increased in short time of with low calcium intake[39], but when long term deficiency of vitamin D or in aged people, serum 1,25-dihydroxyvitamin D level fall with concomitant reduction in intestinal calcium absorption[40,41]. This study found that long term drinking low mineral water decreased the serum 1,25-dihydroxyvitamin D level.

Dahl C recently reported that compared to drinking Norwegian municipal drinking water with a pH ≥7.0, drinking water with a pH <7.0 increased the risk of forearm fractures in a population-based cohort from Norway [12]. In the present study, although the pH of the tap water and the bottled natural water were approximately 7.5, and the pH values of the mineralized water and the bottled purified water were acidic (approximately 6.75). All of the parameters for alkali load (PCO_2,_ HCO_3_^-^act, HCO_3_^-^std, BE (B)], BE (ecf) and ct CO_2_) were the highest in the bNW group and the second highest in the TW group, but there was no statistically significant difference among the four groups (data not show). Although sodium was higher in TW and bNW group than bPW and bMW group, the highest concentrate was 12.4 mg/L which is in tap water. In term of the global goal set by WHO is to reduce salt intake less than 5 g (2000 mg of sodium) per person per day by 2025, and the dose of sodium in most studies that increased calcium excretion were much higher (80g/kg diet or more) than this level [42], and sodium is not the main element of bone as calcium and magnesium. The difference of sodium among these four type water would not mainly influence the health of bone.

In summary, the current study showed that multi-generational drinking of bottled low mineral water (Especially in bottled mineralized water and bottled purified water) impairs bone quality in female rats. Drinking tap water, which contains adequate minerals, was found to be better for bone health. Considering the rapid increase in of bottled water consumption, further studies on the health effects in humans are needed.

## Supporting Information

S1 Table Ion characteristics of the four types of water. All tested parameters in these four water were met the water quality standard (GB/T5750-2006 and GB19298-2003, China). Although arsenic, fluoride and nitrate in tap water were higher than that in other three water, the level of them were much lower than standard limit. TW: Tap water, bNW: bottled natural water, bMW: bottled mineralized water, bPW: bottled purified water.
(DOC)
