# Supplementary material for: Multi-Generational Drinking of Bottled Low Mineral Water Impairs Bone Quality in Female Rats
Source: PLoS One. 2015 Mar 24;10(3):e0121995. doi: 10.1371/journal.pone.0121995 (PMC4372292; doi:10.1371/journal.pone.0121995)
Supplement: S1 Table — All tested parameters in these four water were met the water quality standard (GB/T5750-2006 and GB19298-2003, China). Although arsenic, fluoride and nitrate in tap water were higher than that in other three water, the level of them were much lower than standard limit. TW: Tap water, bNW: bottled natural water, bMW: bottled mineralized water, bPW: bottled purified water. (DOC) [file pone.0121995.s001.doc]

Table S1. Characteristics of the four type water

| **Parameters** | **TW** | **bNW** | **bMW** | **bPW** | **Drinking water quality standard (GB/T5750-2006)** | **Bottled water quality standard (GB19298-2003)** |
| --- | --- | --- | --- | --- | --- | --- |
| Total coliform  (CFU/100mL) | 0 | 0 | 0 | 0 | 0 | ≤3 |
| Thermotolerant coliform organisms (CFU/100mL) | 0 | 0 | 0 | 0 | 0 | -- |
| Total colony count (CFU/mL) | 0 | 0 | 1 | 0 | ＜100 | ≤20 |
| Arsenic（mg/L） | 0.01 | ＜0.01 | ＜0.01 | ＜0.01 | ＜0.01 | ≤0.01 |
| Cadmium（mg/L） | ＜0.001 | ＜0.001 | ＜0.001 | ＜0.001 | ＜0.005 | -- |
| Hexavalent chromium（mg/L） | ＜0.004 | ＜0.004 | ＜0.004 | ＜0.004 | ＜0.05 | -- |
| Lead（mg/L） | ＜0.005 | ＜0.005 | ＜0.005 | ＜0.005 | ＜0.01 | ≤0.01 |
| Mercury（mg/L） | 0.0003 | ＜0.0001 | 0.0002 | ＜0.0001 | ＜0.001 | -- |
| Selenium（mg/L） | ＜0.001 | ＜0.001 | ＜0.001 | ＜0.001 | ＜0.01 | -- |
| Cyanide（mg/L） | ＜0.001 | ＜0.001 | ＜0.001 | ＜0.001 | ＜0.05 | ≤0.002 |
| Fluoride（mg/L） | 0.2 | ＜0.1 | ＜0.1 | ＜0.1 | ＜1.0 | -- |
| Nitrate（N）（mg/L） | 1.2 | 0.5 | ＜0.5 | ＜0.5 | ＜10 | -- |
| Nitrite（mg/L） | ＜0.001 | 0.001 | ＜0.001 | 0.002 | -- | ≤0.002 |
| Trichloromethane(mg/L) | 0.029 | 0.015 | 0.033 | 0.021 | ＜0.06 | ≤0.02 |
| Carbon tetrachloride (mg/L) | ＜0.0001 | 0.0011 | 0.0009 | 0.0013 | ＜0.002 | ≤0.001 |
| Colour | 3 | 3 | 3 | 3 | ＜15 | ≤5 |
| Turbidity（NTU） | 0.16 | 0.01 | 0.01 | 0.01 | ＜1 | ≤1 |
| Odour and taste | No | No | No | No | No unpleasant odour and taste | |
| Visible substance | No | No | No | No | No | No |
| pH value | 7.57 | 7.55 | 6.80 | 6.80 | 6.5～8.5 | 5.0～7.0 |
| Aluminum（mg/L） | ＜0.10 | ＜0.10 | ＜0.10 | ＜0.10 | ＜0.2 | -- |
| Iron（mg/L） | 0.13 | ＜0.01 | ＜0.01 | ＜0.01 | ＜0.3 | -- |
| Manganese（mg/L） | 0.02 | 0.02 | 0.03 | 0.02 | ＜0.1 | -- |
| Copper（mg/L） | 0.05 | 0.06 | 0.05 | 0.05 | ＜1.0 | ≤1.0 |
| Zinc（mg/L） | 0.07 | 0.01 | 0.01 | 0.01 | ＜1.0 | -- |
| Chloride（mg/L） | 16.0 | 5.0 | 5.0 | 2.0 | ＜250 | ≤6.0 |
| Sulfate（mg/L） | 48.2 | 4.4 | 3.8 | 7.8 | ＜250 | -- |
| Total dissolved solids（mg/L） | 229 | 87.2 | 10.9 | 1.2 | ＜1000 | -- |
| Total hardness（CaCO_3_）（mg/L） | 200.3 | 69.6 | 2.3 | 0.8 | ＜450 | -- |
| Oxygen demand（COD_Mn_）（mg/L） | 1.0 | 0.6 | 0.6 | 0.5 | ＜3 | ≤1.0 |
| Volatile phenol（mg/L） | ＜0.001 | ＜0.001 | ＜0.001 | ＜0.001 | ＜0.002 | ≤0.002 |
| Calcium（mg/L） | 52.9 | 10.6 | 0.02 | 0.04 | -- | -- |
| Magnesium（mg/L） | 12.7 | 9.4 | 0.4 | 0.02 | -- | -- |
| Potassium（mg/L） | 2.5 | 3.8 | 3.4 | ＜0.5 | -- | -- |
| Sodium（mg/L） | 12.4 | 9.0 | 0.1 | 0.1 | ＜200 | -- |
| Free chlorine（mg/L） | 0.05 | 0.00 | 0.00 | 0.00 | ＞0.05 | ≤0.005 |

Note: -- means no standard parameter.
